# The Prevalence of Workaholism: A Survey Study in a Nationally Representative Sample of Norwegian Employees

**DOI:** 10.1371/journal.pone.0102446

**Published:** 2014-08-13

**Authors:** Cecilie Schou Andreassen, Mark D. Griffiths, Jørn Hetland, Luca Kravina, Fredrik Jensen, Ståle Pallesen

**Affiliations:** 1 Department of Psychosocial Science, University of Bergen, Bergen, Norway; 2 The Competence Center, Bergen Clinics Foundation, Bergen, Norway; 3 Psychology Division, Nottingham Trent University, Nottingham, United Kingdom; 4 Department FISPPA - Section of Applied Psychology, University of Padova, Padova, Italy; Tilburg University, Netherlands

## Abstract

Workaholism has become an increasingly popular area for empirical study. However, most studies examining the prevalence of workaholism have used non-representative samples and measures with poorly defined cut-off scores. To overcome these methodological limitations, a nationally representative survey among employees in Norway (N = 1,124) was conducted. Questions relating to gender, age, marital status, caretaker responsibility for children, percentage of full-time equivalent, and educational level were asked. Workaholism was assessed by the use of a psychometrically validated instrument (i.e., Bergen Work Addiction Scale). Personality was assessed using the Mini-International Personality Item Pool. Results showed that the prevalence of workaholism was 8.3% (95% CI  = 6.7–9.9%). An adjusted logistic regression analysis showed that workaholism was negatively related to age and positively related to the personality dimensions agreeableness, neuroticism, and intellect/imagination. Implications for these findings are discussed.

## Introduction

Over the last few years, workaholism has become an increasingly studied area for empirical investigation. The construct has been defined in different ways, approached both as an attitude, trait, behavior, compulsion, and/or obsession. The parallels between workaholism and substance addiction have been drawn by scholars for decades [1]. However, this has been controversial despite a number of scholars emphasizing the obsessive-compulsive aspect of this behavior [2]. In line with an addiction approach, Andreassen, Hetland, and Pallesen [3] defined workaholism as “being overly concerned about work, to be driven by strong and uncontrollable work motivation, and to spend so much energy and effort into work that it impairs private relationships, spare-time activities and/or health” (p. 8). Although some researchers have noted certain positive aspects of workaholism, encompassing such aspects as high work motivation [4], [5], the prevailing perspective today suggests that workaholism primarily comprises negative consequences, characterized by compulsiveness and rigidity [1], [2], [6], [7].

There has been some debate about the dimensionality of the construct. For instance, Spence and Robbins [8] argued that workaholism comprises high levels of work involvement, high levels of work drive, and low levels of work enjoyment. Through factor analysis they identified two types of workaholics: enthusiastic workaholics characterized by high scores on all three dimensions; and non-enthusiastic workaholics with high scores on work involvement and drive, but low scores on work enjoyment. The latter type was defined as a “real” workaholic. However, this multidimensional perspective has been subjected to much critique. Empirically, the work involvement dimension has repeatedly failed to show adequate validity [3], [9]–[12], and the work enjoyment dimension has been deemed by some authors as irrelevant in relation to the construct of workaholism [7], [13]. This suggests that the core aspect of workaholism is the compulsive drive and need to work. However, more recent approaches have regained interest for the addiction perspective when it comes to workaholism and define it according to general criteria for addiction [14]–[16], often referring to the following: (1) *salience* (i.e., preoccupation with work), (2) *mood modification* (i.e., work to escape or avoid dysphoria), (3) *conflict* (i.e., work comes in conflict with one's own and others' needs), (4) *withdrawal* (i.e., dysphoria when prohibited from working), (5) *tolerance* (i.e., work increasingly more to achieve the same mental and physiological effect), (6) *relapse* (i.e., falls back into old pattern after a period of improvement), and (7) *problems* (i.e., work so much that health, relationships, hobbies, etc. are negatively affected) [15], [17], [18]. Withdrawal and tolerance covers what is normally understood as dependence [19], thus addiction is a wider concept covering all the seven components referred to above. The seven components are further in line with diagnostic addiction criteria employed in current diagnostic taxonomies [20]–[22].

Although concepts of “workaholism” and “work addiction” have been used interchangeably, most researchers have not defined and measured the concept in line with the aforementioned criteria. Previous instruments, such as the Workaholism Battery (WorkBAT) [8], the Work Addiction Risk Test (WART) [23], and the Dutch Work Addiction Scale (DUWAS) [7] conceptualize workaholism as either an attitude [8], obsession-compulsion [7], or as a Type-A behavior [23]. More recently, the Bergen Work Addiction Scale (BWAS) was constructed [15] specifically based on Brown's [17] behavioral addiction components and on Griffiths' [18] components model of addiction. All items in the BWAS are scored along a 5-point Likert scale ranging from ‘never’ (1) to ‘always’ (5) asking how often during the last year the symptoms have occurred. Endorsing ‘often’ or ‘always’ on four (or more) out of seven criteria indicates workaholism. This cut-off was set in accordance with previous operationalizations of behavioral addiction [24] as well as with the nosological approach found in contemporary psychiatric diagnostic systems [21], [22].

Additionally, the suggested cut-off for categorization of workaholics demonstrated good discriminative ability with respect to working hours, leadership position, and subjective health complaints in the initial construction of the scale. This cut-off procedure has also been confirmed in analyses among 701 Italian workers (378 females), where the suggested cut-off discriminated between workaholics and non-workaholics in terms of working hours and levels of exhaustion, showing that workaholics worked significantly more and perceived more exhaustion than non-workaholics [25]. The BWAS score has been found to be positively associated with scores on the DUWAS (r = .55–.58), WART (r = .50–.84) and WorkBAT (r = .35–.65), but appears less related to the WorkBAT-Enjoyment subscale (r = .13). In addition, Molino [25] found support for the factor structure of BWAS. Construct validity was confirmed when investigating the correlations with other relevant workaholism constructs such as job satisfaction (r = −.15), work-family conflict (r = .59), workload (r = .45), cognitive demand (r = .22), emotional demand (r = .22), and emotional dissonance (r = .34). A diary study (comprising 96 individuals) and a multilevel research design was also performed, confirming the within-person variations and reliability (α = .79−.87) of BWAS over time [25]. A more recently developed instrument is the 28-item Work Craving Scale (WCS) [16] – also rooted in the addiction paradigm. The developers of this scale integrated theory and research on craving and workaholism and found – in line with their predictions – support for four dimensions of work craving: (i) obsessive-compulsive desire for work, (ii) anticipation of self-worth compensation, (iii) anticipation of reduction of negative affect or withdrawal symptoms resulting from working, and (iv) neurotic perfectionism [16].

In terms of health and psychosocial impact or correlates, several studies have attested to the positive associations between workaholism and work-family conflicts [11], [26]–[29] and between workaholism and subjective stress-related somatic and psychological symptoms [8], [9], [11], [28], [30]–[32]. In relation to personality factors, the Five-Factor Model of personality [33], workaholism appears to be positively related to neuroticism (e.g., being nervous and sensitive) [34]–[36] and conscientiousness (e.g., being organized and efficient) [34], [35], [37], and negatively related to agreeableness (e.g., being sympathetic and friendly) [34] and openness (e.g., being imaginative and inventive) [35]. However, one study reported workaholism to be positively related to openness [37]. Other studies have shown that workaholism appears to be unrelated to gender but associated with age [34], [38] with younger adults being more likely to be workaholics.

To date, estimates of the prevalence of workaholism have varied greatly depending upon the samples surveyed and the instruments used. Studies conducted among students report prevalence rates ranging from 14% (as measured by Shorter PROMIS Questionnaire) [39] to 18% (self-identified) [40]. In a small survey study of 219 adults, Spence and Robbins [8] reported a workaholism prevalence of 8% among men and 13% among women (as measured by WorkBAT). In a sample of 192 attorneys, physicians and psychologists/therapists, a prevalence rate of 23% (based on an adapted version of a questionnaire developed by Machlowitz [4]) was reported [41]. However, it should be noted that Machlowitz [4] primarily regarded workaholism as a positive entity. In another study, Kanai et al. [9] found a prevalence of 21% among 962 male Japanese workers (as measured by WorkBAT). Based on a sample of 519 Canadian university business graduates, a prevalence rate of 13% for workaholism (as measured by WorkBAT) was reported [42]. Based on the workaholism literature as a whole, some arguably crude and general estimates not anchored in any specific study about the prevalence of workaholism in the general population have been put forward, ranging from 5% [4] to 10% [43], and even up to 25% [6].

One of the major limitations with all of the aforementioned studies is that none of them carried out their research using a nationally representative sample of workers. Additionally, most previously published studies have relied (at least in part) on assessment of dimensions that are irrelevant for workaholism (e.g., work enjoyment) and have used instruments not based on addiction criteria. Furthermore, the majority of workaholism measures used in such studies failed to employ well-defined cut-off scores for categorizing workaholism. Although the relationships between workaholism and other variables such as personality traits has been established in prior research, there still are some discrepancies across studies, thus deserving further investigation.

Given the conceptual, theoretical, and methodological limitations of almost all previous empirical research, a study based on a representative national sample of Norwegian workers administering a validated workaholism measure (BWAS) [15] with specific cut-off scores in order to assess the prevalence of workaholism was conducted. A further aim of the present study was to investigate relevant associations between workaholism, socio-demographics (i.e., age, gender, marital status, children living at home, education, and percentage of full-time equivalent) and personality (based on the Five-Factor model of personality). Based on prior research it was hypothesized that workaholism would be positively related to neuroticism and conscientiousness, negatively related to agreeableness and openness, and unrelated to extraversion. Furthermore, it was expected that workaholism would be unrelated to gender, but related to age (i.e., younger adults being more likely to experience workaholism).

## Methods

### Sample and procedure

A sample of 2,160 participants, aged 18–70 years old, was randomly drawn from the AA-registry of Norway (i.e., a central registry of all employers and employees in Norway) during January to June 2012. According to Norwegian legislation, the National Insurance Act (§-25-1), it is mandatory for all employers to register workers being hired in the AA-registry when the employment lasts for more than seven days and comprise four hours or more of work per week. All employees in the sample received a letter stating that they would be receiving a questionnaire about attitudes towards work via postal mail. A four-page questionnaire was sent four days later together with a pre-paid return envelope and an information letter, where it was emphasized that participation was voluntarily. Consent to participate was deemed given upon completion and return of the questionnaire. The information letter also contained a web address that when accessed gave the respondents the opportunity to answer the questionnaire online. In the information letter, the potential responders were informed that of those who participated, 50 would be randomly drawn to receive a voucher with a value of 500 NOK (approximately 80 US$).

One reminder letter was sent to those who did not respond during the first month after the questionnaire was sent. A total of 44 letters were returned due to wrong address and 34 participants were not working for various reasons (e.g., long-term sickness, retirement) leaving a total of 2,082 participants eligible for study participation. Of these, a total of 1,124 responded, yielding a response rate of 54.0%. Of those who responded, 1,013 completed the paper version of the questionnaire, whereas 111 completed the survey online. A total of 967 initially responded following the first mailing. A further 157 participants responded following the reminder letter. In order to adjust for discrepancies between the initial sample and the final sample, the following weights were calculated and employed: 1.71 (males 18–31 years), 1.10 (males 32–45 years), 0.87 (males 46–58 years), 0.79 (males 59–70 years), 1.30 (females 18–31 years), 0.92 (females 32–45 years), 0.78 (females 46–58 years) and 0.93 (females 59–70 years). The study was approved by the Norwegian Social Science Data Services (No. 28071) and by the Norwegian Labour and Welfare Service. Data relating responses to the questionnaire and data concerning names and addresses were kept separately. The latter personal information was kept locked in approved cabinets and was deleted upon completion of the study.

### Instruments

#### Socio-demographics

Questions relating to gender, age, marital status, caretaker responsibility for children living at home, percentage of full-time equivalent, and education were asked.

#### The Bergen Work Addiction Scale (BWAS)

The BWAS was used to assess work addiction. This scale comprises seven workaholism items/symptoms using addiction criteria (i.e., salience, tolerance, mood modification, relapse, withdrawal, conflict, and problems) experienced during the past year. Items are worded in line with diagnostic criteria for addiction [20], [22]. Each item is answered on a 5-point Likert scale ranging from ‘never’ (1) to ‘always’ (5). The statistical methodology and empirical underpinnings in the scale-construction study [15] involved item selection analysis, confirmatory factor analysis (RMSEA  = 0.08, CFI  = .96, TLI  = .95), analysis of internal consistency (α = .84/.80), as well as cross-validation against relevant constructs. The analyses were based on data from a sample of 12,137 Norwegian employees representing several professions. A score of 4 (‘often’) or 5 (‘always’) on at least four of the seven items was recommended as a cut-off for workaholism being present in that individual (i.e., a polythetic approach in line with modern psychiatric nosology). The cut-off was validated against criteria such as working hours and leadership responsibilities, and demonstrated good discriminative ability. Cronbach's alpha for the BWAS in the present study was .81.

#### The Mini-International Personality Item Pool (Mini-IPIP)

The Mini-IPIP was used as a measure of the Five-Factor Model of personality. The Mini-IPIP comprises 20 items, four reflecting each of the following five dimensions: *extraversion* (e.g., being outgoing, talkative), *agreeableness* (e.g., being sympathetic and warm), *conscientiousness* (e.g., being organized and structured), *neuroticism* (e.g., being nervous and moody), and *intellect/imagination* (e.g., being creative and intellectual), the latter being equal to the openness dimension. Each item is answered on a five-point Likert scale ranging from ‘very inaccurate’ (1) to ‘very accurate’ (5) [44]. The Cronbach's alphas for the five subscale of the Mini-IPIP in the present study were .78, .75, .66, .66, and .67, respectively.

### Statistics

Descriptive statistics in terms of distribution of nominal variables and in terms of means and standard deviations for variables measured on interval/ratio scales were calculated in order to characterize the sample. The prevalence of workaholism (with 95% confidence interval; 95% CI) was calculated. For this estimate, the data were weighted in order to adjust for the age and gender discrepancy between the initial sample and the final sample. In order to investigate factors associated with workaholism a correlation analysis was conducted analyzing the association between the sum score of the BWAS, the sum scores of the five Mini-IPIP-dimensions and age. Further, the sum score of the BWAS was compared across different levels of the nominal variables (gender, marital status, caretaker responsibility for children, percentage of full-time equivalent and education) by t-tests for independent samples or by one-way ANOVA.

Additionally, logistic regression analyses were conducted, where workaholism (0 =  not workaholic, 1 =  workaholic) in accordance with the given cut-off comprised the dependent variable. The independent variables comprised gender, age group (18–31 years, 32–45 years, 46–58 years and 59–70 years), marital status (not living with a partner vs. living with a partner), caretaker responsibility for children (no/yes), percentage of full-time equivalent (less than 100% vs. 100% or more), education (compulsory school, high school, vocational school/technical college, bachelor's degree, master's degree, and PhD), and the five personality dimensions (extraversion, agreeableness, conscientiousness, neuroticism, and intellect/imagination). Age groups and education levels were dummy coded.

Both crude analyses and an adjusted logistic regression analysis were conducted. The notions of ‘crude’ and ‘adjusted’ are commonly used when describing results from logistic regression analyses. The ‘crude’ results represent the bivariate associations (between the independent and dependent variable), whereas ‘adjusted’ results reflect the multivariate association (between each of the independent variables on one side, and the dependent variable on the other, controlled for all other independent variables). In the crude analyses, each of the independent variables was entered separately, investigating the bivariate relationship between the independent and the dependent variable. In the adjusted analysis, all the independent variables were entered simultaneously, investigating the multivariate associations between the independent variables and the dependent variable. The odds ratio (OR) can be regarded as significant when the 95% CI does not include 1.00. Similar to the estimate of the workaholism prevalence, the logistic regression calculations were also weighted in order to adjust for the age and gender discrepancy between the initial sample and the final sample. The dataset is available as a Data S1.

## Results

Table 1 shows descriptive data of the sample. The prevalence of workaholism in the current sample was estimated to 8.3% (95% CI  = 6.7–9.9%). No difference in prevalence rate of workaholism was detected between those answering the paper-based and web-based survey (χ^2^ = 0.0, df  = 1, *p* = .99, continuity corrected). A sensitivity analysis concerning different cut-offs (scoring 4 or 5 on 1 to 7 items) revealed workaholism prevalence rates ranging from 46.6% (scoring 4 or 5 on one item only) to 0.3% (scoring 4 or 5 on all seven item). The results of sensitivity analysis are presented in Table 2.

**Table 1:** Descriptive data for the sample (N = 1,124).

Variable		Percentage	Mean (SD)
**Gender**	Male	49.0%	
	Female	51.0%	
**Age group**	18–31 years	15.7%	
	32–45 years	32.6%	
	46–58 years	36.5%	
	59–70 years	15.3%	
**Marital status**	Not living with a partner	17.5%	
	Living with a partner	82.5%	
**Childcare responsibility**	No	57.0%	
	Yes	43.0%	
**Full-time equivalent**	Less than 100%	21.7%	
	100% or more	78.3%	
**Education**	Compulsory school	7.8%	
	High school	10.2%	
	Vocational school	33.7%	
	Bachelor's degree	31.7%	
	Master's degree	14.5%	
	PhD	2.2%	
**Personality**	Extraversion		14.08 (3.50)
	Agreeableness		16.96 (2.71)
	Conscientiousness		16.56 (2.74)
	Neuroticism		10.62 (3.32)
	Intellect/Imagination		13.99 (3.24)

**Table 2:** Sensitivity for different cut-offs based on the Bergen Work Addiction Scale (N = 1,108).

Number of items with score of 4 (often) or 5 (always)	Estimated prevalence	95% Confidence interval
1 item	46.6%	43.6–49.5%
2 items	27.7%	25.1–30.4%
3 items	14.8%	12.7–16.9%
4 items	8.3%	6.7–9.9%
5 items	4.6%	3.3–5.8%
6 items	1.7%	0.9–2.4%
7 items	0.3%	−0.1–0.7%

Table 3 presents the percentage of those endorsing each of the seven workaholism criteria of the BWAS (i.e., scoring 4 or 5). This varied from 6.4% (BWAS Item 3) to 30.5% (BWAS Item 2). Table 4 provides the correlation coefficients between all the study variables. The sum score of the BWAS correlated significantly and inversely with age, and significantly positively with caretaker responsibility, full time equivalent, educational level, the sum score of Extroversion, Agreeableness, Neuroticism and Intellect/Imagination and significantly and negatively with age and the sum score of Conscientiousness. Table 5 shows the comparison of the mean score of the BWAS across the levels of nominal variables (gender, marital status, caretaker responsibility for children, percentage of full-time equivalent and education). The weighted mean score of the BWAS for the whole sample was 15.49 (SD  = 4.93). No mean score difference was found for gender or marital status. Those with caretaker responsibility for children had a higher mean score than those without childcare responsibilities. In terms of education, those with compulsory school scored lower than those with college/university degrees (bachelor, master, and PhD). Respondents with vocational school/technical college had lower scores on the BWAS compared to those with master and PhD levels from colleges/universities.

**Table 3:** Percentage and 95% confidence interval (95% CI) of the respondents who endorsed (scoring 4 or 5) on the items of the Bergen Work Addiction Scale (BWAS; Andreassen, Griffiths, et al., 2012) (N = 1,108).

Item	Wording	Addiction component	Percentage (95% CI) scoring 4 or 5
BWAS1	Thought of how you could free up more time to work?	Salience	10.4% (8.2–12.2%)
BWAS2	Spent much more time working than initially intended?	Tolerance	30.5% (27.7–33.2%)
BWAS3	Worked in order to reduce feelings of guilt, anxiety, helplessness and/or depression?	Mood modification	6.4% (5.0–7.9%)
BWAS4	Been told by others to cut down on work without listening to them?	Relapse	8.0% (6.4–9.6%)
BWAS5	Become stressed if you have been prohibited from working?	Withdrawal	12.3% (10.4–14.2%)
BWAS6	Deprioritized hobbies, leisure activities, and/or exercise because of your work?	Conflict	24.6% (22.0–27.1%)
BWAS7	Worked so much that is has negatively influenced your health?	Problems	11.8% (9.8–13.7%)

**Table 4:** Correlation coefficients (Pearson's moment-product correlation coefficients, point-biserial correlation coefficients and Phi coefficients between all study variables (Bergen Work Addiction Scale (BWAS), gender, age group, marital status, caretaker responsibility for children, percentage of full-time equivalent, education and the five dimensions of the Mini-International Personality Item Pool (Extraversion, Agreeableness, Conscientiousness, Neuroticism, Intellect/Imagination)) (N = 1,107–1,124).

Variable		2	3	4	5	6	7	8	9	10	11	12	13	14	15	16	17	18	19	20
**1.**	**BWAS**	.02	.08*	.10**	−.08*	−.13**	−.03	.12**	.08**	−.11**	.01	−.07*	.04	.09**	.08**	.08**	.07*	−.17**	.26**	.13**
**2.**	**Gender^a^**		.04	−.02	−.04	.03	.03	.03	.27**	.04	−.02	.10**	−.09**	−.02	−.01	−.01	−.30**	−.20**	−.14**	.15**
**3.**	**18–31 years**			−.40**	−.37**	−.23**	−.18**	−.20**	−.10**	−.00	.12**	−.01	.02	−.08**	−.08**	.07*	−.03	−.01	.05	.02
**4.**	**32–46 years**				−.45**	−.28**	.08*	.46**	.11**	−.02	−.04	−.10**	.05	.12**	−.00	.04	.03	−.08**	.03	.05
**5.**	**46–58 years**					−.25**	.06*	−.05	.06*	−.00	−.06*	.09**	−.06*	−.02	.08**	−.05	.03	.10**	−.05	−.02
**6.**	**59–70 years**						.04	−.31**	−.10**	.03	−.02	.03	−.01	−.03	−.01	−.08**	−.04	−.01	−.04	−.07*
**7.**	**Marital status^b^**							.24**	.07*	−.07*	−.09**	.00	.06*	.03	.03	.01	.01	.05	−.02	−.01
**8.**	**Childcare^c^**								.11**	.01	−.06*	−.09**	.08**	.04	.05	.04	.05	−.04	.02	.05
**9.**	**Full-time equivalent^d^**									.08**	−.09**	−.01	.02	.12**	.05	.08**	−.07*	−.01	−.13**	.07*
**10.**	**Compulsory school**										−.10**	−.21**	−.20**	−.12**	−.04	−.10**	−.06*	−.06*	.05	−.12**
**11.**	**High school**											−.25**	−.24**	−.14**	−.05	.02	.04	.04	.03	.02
**12.**	**Vocational school**												−.48**	−.29**	−.10**	−.04	−.10**	.02	.04	−.12**
**13.**	**Bachelor's degree**													−.28**	−.10**	.06	.08**	.00	−.05	.04
**14.**	**Master's degree**														−.06	.04	.03	.00	−.05	.13**
**15.**	**PhD**															−.01	.04	−.05	−.01	.12**
**16.**	**Extraversion**																.35**	.08**	−.08**	.26**
**17.**	**Agreeableness**																	.17**	.00	.17**
**18.**	**Conscientiousness**																		−.16**	−.06*
**19.**	**Neuroticism**																			−.06*
**20.**	**Intellect/Imagination**																			–

*p<.05,

**p<.01, a) female  = 1, male  = 2, b) not living with a partner  = 1, living with a partner  = 2, c) no caretaker responsibility for children  = 1, caretaker responsibility for children  = 2, d) less than 100%  = 1, 100% or more  = 2.

**Table 5:** Comparisons of mean scores of the Bergen Work Addiction Scale (BWAS) across different levels of nominal variables (gender, marital status, caretaker responsibility for children, percentage of full-time equivalent (EQV) and education) (N = 1,122).

Variable		Mean	SD	Statistics
**Gender**	Male (n = 554)	15.44	4.99	t = 0.5, df = 1120, *p* = .61
	Female (n = 568)	15.30	4.81	
**Marital status**	Not living with a partner (n = 195)	15.66	4.90	t = 1.0, df = 1111, *p* = .34
	Living with a partner (n = 918)	15.29	4.89	
**Childcare**	No (n = 634)	14.82	4.83	t = 4.3, df = 1108, *p*<.001
	Yes (n = 476)	16.10	4.89	
**Full-time EQV**	Less than 100% (n = 241)	14.50	4.48	t = 3.4, df = 1110, *p*<.005
	100% or more (n = 871)	15.64	4.99	
**Education**	1. Compulsory school (n = 65)	13.39	4.47	F_5,1108_ = 8.0, *p*<.001
	2. High school (n = 111)	15.29	4.87	
	3. Vocational school (n = 376)	14.80	4.85	Post hoc (Bonferroni):
	4. Bachelor's degree (n = 353)	15.67	4.95	1<4, 5, 6 (all *p*<.05)
	5. Master's degree (n = 164)	16.53	4.71	3<5, 6 (all *p*<.05)
	6. PhD (n = 25)	18.36	3.80	

Table 6 presents the results from the logistic regression analyses in terms of odds ratios (OR) and 95% confidence intervals (95% CI) for both the crude and the adjusted analyses. For the dummy coded variables (i.e., age groups and education), the largest group comprises the reference (for which the OR is set to 1.00). In both the crude and adjusted analyses, workaholism was positively associated with those aged 18–31 years and 32–45 years, compared to the contrast group (46–58 years). Having caretaker responsibility for children was significantly associated with workaholism in the crude analysis, but did not remain significant in the adjusted analysis. In relation to personality, workaholism was unrelated to agreeableness in the crude analysis. When adjusting for all the other independent variables a significant and positive relationship between workaholism and agreeableness was revealed. Conscientiousness was significantly and negatively associated with workaholism in the crude analysis, but this relationship was no longer significant when controlling for other variables in the adjusted analysis. Neuroticism and intellect/imagination was positively and significantly associated with workaholism in both the crude and in the adjusted analyses. The full model containing all predictors (adjusted analysis) was statistically significant (χ^2^ = 60.9, df  = 17, *p*<.01). Furthermore, the model as a whole explained between 5.7% (Cox and Snell R square) and 13.5% (Nagelkerke R squared) of the variance in workaholism status and correctly classified 92.2% of all cases. The proportion of corrected classified cases did not increase from the null model.

**Table 6:** Logistic regression analysis, where workaholism (0 =  not workaholic, 1 =  workaholic) comprised the dependent variable and where gender, age, marital status, caretaker responsibility for children, percentage of full-time equivalent, education and personality comprised the independent variables (N = 1,044).

Variable		Crude OR	Adjusted OR
		(95% CI)	(95% CI)
**Gender**	Female	1.00	1.00
	Male	1.20 (0.78–1.85)	1.61 (0.93–2.81)
**Age group**	46–58 years	1.00	1.00
	18–31 years	2.06 (1.08–3.94)	2.11 (1.02–4.37)
	32–45 years	2.52 (1.39–4.56)	2.01 (1.02–3.98)
	59–70 years	1.32 (0.57–3.06)	1.66 (0.64–4.31)
**Marital status**	Not living with a partner	1.00	1.00
	Living with a partner	0.91 (0.53–1.58)	1.03 (0.53–1.99)
**Childcare responsibility**	No	1.00	1.00
	Yes	1.57 (1.02–2.43)	1.25 (0.70–2.23)
**Full-time equivalent**	Less than 100%	1.00	1.00
	100% or more	1.61 (0.89–2.91)	1.36 (0.70–2.64)
**Education**	Vocational school	1.00	1.00
	Compulsory school	0.44 (0.12–1.56)	0.31 (0.07–1.45)
	High school	1.28 (0.61–2.70)	1.14 (0.49–2.64)
	Bachelor's degree	1.34 (0.78–2.28)	1.37 (0.76–2.48)
	Master's degree	1.46 (0.76–2.82)	1.23 (0.58–2.60)
	PhD	1.69 (0.42–6.71)	1.25 (0.28–5.65)
**Personality**	Extraversion	1.01 (0.94–1.07)	0.98 (0.91–1.06)
	Agreeableness	1.08 (0.99–1.18)	1.12 (1.00–1.25)
	Conscientiousness	0.88 (0.82–0.95)	0.93 (0.85–1.01)
	Neuroticism	1.20 (1.12–1.28)	1.21 (1.13–1.31)
	Intellect/Imagination	1.11 (1.03–1.19)	1.09 (1.01–1.19)

## Discussion

The main aim of the present study was to estimate the prevalence of workaholism (assessed here as a behavioral addiction by BWAS) in a nationally representative sample of Norwegian employees. Based on the results of the survey, the prevalence of workaholism was estimated to be 8.3%. Due to the many aforementioned methodological shortcomings of previous workaholism research, the estimate in the present study is not directly comparable to the prevalence reported by others, but appears similar to the 10% estimate presented in a recent comprehensive review [43]. The sensitivity analysis revealed the prevalence rates of workaholism ranging from 0.3% to 46.6% depending of the cut-off employed. In the present study an endorsement of at least 4 of 7 items as the cut-off was used, and is in line with the authors' previous suggestion [15]. The fact that more than 8% of the working population appears to suffer from workaholism underlines the need for proper treatment and other relevant interventions. Although several therapies have been suggested, such as the 12-step program of Workaholic Anonymous [45], motivational interviewing [46], cognitive-behavioral therapy [47], family therapy [48], positive psychology [49], recovery training [50], organizational based interventions [38], [51], and meditation awareness training [52], no well controlled study of treatment of workaholism, to the best of the authors' knowledge, has so far been conducted and/or published. The evidence suggests a relatively high prevalence of workaholism, but no empirically validated treatment effectiveness study currently exists.

The prevalence endorsing (scoring 4 or 5) the different items of the BWAS was also calculated. The prevalence rates ranged from 6.4% to 30.5%. One could argue that this suggests that different weights should be allocated to the different symptoms. However, such an approach is not in line with modern psychiatric nosology, where typically a certain number of symptoms, irrespective of their prevalence, needs to be present in order to make a diagnosis [21]. It is also common not to weight the different items when using clinical scales for assessing the prevalence of common disorders, such as depression and anxiety [53]–[55]. However, it may be of both theoretical and applied interest to subject workaholism measures to Rasch modeling, that places people and symptoms on the same latent scale or metric. This would enable the level of workaholism associated with a specific symptom and the overall level of workaholism of individuals to be compared directly. Such analyses have been conducted for assessment of other non-chemical addictions [56], but (to date) has not been done with workaholism instruments. This topic touches upon the debate related to whether psychopathology best can be understood as discrete entities in line with current diagnostic systems or if psychopathology best is understood along a continuum [57].

Gender was not found to be related to workaholism, neither in terms of mean differences nor in terms of the results from the logistic regression analysis. This is in line with the present authors' hypothesis as well as several previous studies [34], [38], [42] although a male preponderance has been reported by some authors [58]. In the present study, workaholism correlated with younger adult age groups and in the logistic regression analysis higher odds ratios for workaholism were found in the age groups 18–31 years and 32–45 years compared to those aged 46–58 years. This corroborates findings from other studies [34], [38] and are also supportive with the claims that the incidence of workaholism is increasing due to societal changes [59]. The findings reported here concerning age might reflect a cohort effect in line with such claims but may also represent an effect of age in itself. The results may also suggest that workaholics over time quit working to a higher degree than non-workaholics. Lower workaholism scores at higher ages may also be explained by the fact that some individuals ‘wise up’ and adjust their work pattern over time because of other commitments (e.g., having a family). It is also possible that workaholics are more at risk of dying early than non-workaholics, and as such the prevalence estimate may have decreased in the higher age group. Longitudinal studies and trend studies are needed in order to clarify these issues. The hypothesis regarding the relationship between workaholism and age was thus supported.

The results of the present study also showed workaholism to be unrelated to marital status and supports some previous studies [60], although workaholism has been related to work-family conflicts [26] and relationship strain [61]. As with marital status, workaholism was not related to caretaker responsibility for children in the adjusted analysis. However, in the crude analysis and in terms of mean score, comparisons people with caretaker responsibilities for children had higher scores and prevalence rates. Although the mean score of the BWAS was higher among people with full-time equivalent compared to those with less than full-time equivalent position, workaholism was unrelated to percentage of full-time equivalent in the logistic regression analysis. This may run counter to some definitions of workaholism [62] and studies showing a positive relationship between work hours and workaholism [63]. However, in the present study participants were asked to provide information about their official percentage of full-time equivalent. This may deviate from the actual time spent working if factors such as overtime are included.

It should also be noted that some people may work for very long hours but not be classified as having problems or being addicted because of other temporary internal factors (e.g., financial problems), external situational factors (e.g., order demand [64]), and/or simply because there are no negative consequences [65]. However, workaholism may also involve thinking about work, even when not actually working [15]. In the present study, people with higher education had higher mean scores on the BWAS than those with lower education. However in the logistic regression analyses education was unrelated to workaholism. This latter is consistent with Porter [66] who hypothesized that workaholism exists across all educational levels [66] and with previous studies [67], [68].

In relation to the Five-Factor Model of personality, agreeableness was positively related to workaholism. This runs counter to the present authors' hypothesis, a previous study [34], and a recent study that also reported that agreeableness was negatively related to several behavioral addictions [69]. People that score high on agreeableness typically emphasize living in harmony with others [70]. As workaholism is related to marital strain and conflicts [71], the finding in the present study regarding agreeableness may seem at odds with what one would expect. However, one might assume that people with high scores on agreeableness are also likely to be sensitive to expectations and wishes from the colleagues and superiors, and this may motivate them to work more compulsively (i.e., the ‘Careaholic workaholics’ [23]), specifically when asked or encouraged to do so by superiors and/or colleagues.

Most previous studies have reported a positive association with workaholism and conscientiousness [34], [35], [37]. However, the correlation coefficient between these two variables in the present study was negative. In the adjusted regression analyses, no relationship between these variables was found. Therefore, the finding regarding conscientiousness was not in line with the proposed hypothesis. Why this was the case in the present study is unclear. However, the workaholism measure used in the present study was based on addiction criteria. Studies of addictions in general do show that they are negatively associated with conscientiousness [72]. This may explain the discrepancy between the present and previous studies in terms of the relationship between workaholism and conscientiousness.

As with earlier studies [34]–[36], workaholism in the present study positively associated with neuroticism, which theoretically resonates well with the notion of workaholism as a compulsive tendency to work excessively [2] and supports our hypothesis. The present study also found that workaholism was positively related to intellect/imagination, a finding that contradicts a previous study and our hypothesis [35] but accords another [37]. One explanation to why workaholism is related to intellect/imagination is that people with high scores on this trait are likely to be intelligent and curious and thus more involved in work. Also, occupations that encourage imaginative thinking can be assumed to initiate work drive in such people [37].

### Strengths and limitations

The present study was conducted in Norway, thus the findings cannot necessary be generalized to workers from other countries. In hindsight, it would have been useful to have examined other specific variables in the survey such as the type of work or level within their organizations, or whether participants were organizationally employed versus self-employed. However, this is not related to the representativeness of the sample *per se* – but rather placing limits on the possible covariates that workaholism may be related to. It should also be noted that the study was cross-sectional, thus no conclusions can be drawn in terms of the directionality and cause-and-effect relationships between study variables. Furthermore, all data were based on self-report. The results may therefore be influenced by the common method bias [73]. In addition, it should be noted that some demographic groups were under-represented in the sample whereas other groups were over-represented. Although this was adjusted for by weighting the data, influence on the results from selection and response bias cannot be ruled out [74]. As no formal diagnostic criteria or gold standard for assessment of workaholism currently exists, the appropriateness of the cut-off score can be further debated. Despite these limitations, there are several key strengths to the present study that deserve mention. The present study is (to the authors' knowledge) the first empirical study to assess workaholism in a nationally representative sample of employees. The sample was selected from the AA-registry and covered the whole of the country. Due to the way the sample was drawn (and the more than acceptable response rate for a national study), it is arguably a nationally representative sample of employees in Norway. Workaholism was assessed with an instrument (i.e., BWAS) that is based on a specific theoretical (addiction) approach [18] and that (unlike most other scales for assessing workaholism) comes with a suggested cut-off for diagnosing/categorization of workaholism [15]. The personality instrument employed (i.e., Mini-IPIP) has also been well-validated [44], although some of its subscales displayed seemingly quite low alphas (.66 and .67), although it should be kept in mind that these scales only contained four items. As noted, the response rate (54.0%) is more than acceptable and arguably higher than many found in national studies. The study comprised 1,124 participants, thus providing high statistical power and that consequently limits the probability of conducting Type 2 errors [75].

### Conclusions

The findings of the present study indicate that workaholism (measured as a behavioral addiction) had a prevalence of 8.3% in a nationally representative sample of Norwegian employees, and that younger adults are more likely to be affected. In terms of personality traits, workaholism is positively related to agreeableness, neuroticism, and intellect/imagination. The results suggest that workaholism is prevalent in a significant minority of those that work and that employers need to encourage employees to work to their contracted hours as overworking in the long run may have deleterious costs on productivity for the organization for which they work (e.g., absenteeism due to ill-health) [76]. The fact that workaholism is rarely treated on a level akin to other more traditional addictions (e.g., alcoholism, gambling addiction, etc.) suggests that problems relating to work may not be conceptualized by those people suffering as something that needs treating. This is further compounded by the fact that work is an activity that society expects people to be typically engaged in eight hours a day. A non-work activity taking up eight hours a day (e.g., gaming, shopping, sex) would typically be pathologized whereas work is not because that is viewed as an activity that people should be doing above and beyond other such activities.

## Supporting Information

Data S1 **The file contains detailed information of all data underlying the findings described in this study.**
(SAV)
